# Knowledge translation of prediction rules: methods to help health professionals understand their trade-offs

**DOI:** 10.1186/s41512-021-00109-3

**Published:** 2021-12-13

**Authors:** K. Hemming, M. Taljaard

**Affiliations:** 1 Institute of Applied Health Research, University of Birmingham, Birmingham, B15 2TT UK; 2 Clinical Epidemiology Program, Ottawa Hospital Research Institute, 1053 Carling Avenue, Ottawa, Ontario K1Y4E9 Canada; 3 School of Epidemiology and Public Health, University of Ottawa, Ottawa, Ontario Canada

**Keywords:** Prediction rules, Population diagrams, Natural frequencies

## Abstract

Clinical prediction models are developed with the ultimate aim of improving patient outcomes, and are often turned into prediction rules (e.g. classifying people as low/high risk using cut-points of predicted risk) at some point during the development stage. Prediction rules often have reasonable ability to either rule-in or rule-out disease (or another event), but rarely both. When a prediction model is intended to be used as a prediction rule, conveying its performance using the C-statistic, the most commonly reported model performance measure, does not provide information on the magnitude of the trade-offs. Yet, it is important that these trade-offs are clear, for example, to health professionals who might implement the prediction rule. This can be viewed as a form of knowledge translation. When communicating information on trade-offs to patients and the public there is a large body of evidence that indicates natural frequencies are most easily understood, and one particularly well-received way of depicting the natural frequency information is to use population diagrams. There is also evidence that health professionals benefit from information presented in this way.

Here we illustrate how the implications of the trade-offs associated with prediction rules can be more readily appreciated when using natural frequencies. We recommend that the reporting of the performance of prediction rules should (1) present information using natural frequencies across a range of cut-points to inform the choice of plausible cut-points and (2) when the prediction rule is recommended for clinical use at a particular cut-point the implications of the trade-offs are communicated using population diagrams. Using two existing prediction rules, we illustrate how these methods offer a means of effectively and transparently communicating essential information about trade-offs associated with prediction rules.

## Making sense of clinical prediction rules: a proposal to aid assessment of clinical utility

Clinical prediction models are developed with the ultimate aim of improving patient outcomes [8, 14]. Prediction models take as inputs various patient characteristics or risk factors (e.g. age, gender, comorbidities) and provide as an output a prediction of the probability of either having or developing a particular disease or outcome (called an “event”), for example, future heart disease, cancer recurrence or lack of response to some treatment. When used to predict the likelihood of having a particular disease they are referred to as diagnostic models, and when used to predict outcomes, they are referred to as prognostic models. Subsequent to model development, prediction models should be internally and externally validated, and then the performance of the prognostic model evaluated in an implementation study—so that impact on clinical outcomes can be determined [16, 29, 31].

There are numerous ways that prediction models can be translated for use in clinical practice. One approach is to formulate a directive decision rule based on cut-points for the predicted probabilities—for example, low or high risk [2, 6, 8]. We refer to this as a prediction rule [25]. Patient care might then be stratified on the basis of these cut-points—and consequently, the model can be thought of as acting like a prediction “rule” [16, 26]. An alternative is to provide individual predicted risks which can be used by the health care professional in guiding therapeutic decisions for individual patients. A recent systematic review found that three quarters of prediction models, in cancer, report associated prediction rules [22]. Here, our focus is on prediction models which are used to risk-stratify patients or recommend treatment or management strategies based on cut-points of predicted risk. Examples of commonly used prediction rules are the Ottawa ankle score [28] and the Framingham heart score [32]. Other examples are the Canadian Syncope Risk Score [30] and the QRISK2 score [4, 5] which we include as case studies (Tables 1 and 3). Although prediction rules can be based on multiple risk strata, for example, low, medium or high risk, for simplicity, we focus on the scenario where predicted probabilities are dichotomised, say, into two groups: low and high risk.

**Table 1:** Case Study 1 the Canadian Syncope Risk Score

**Fig. 1:**
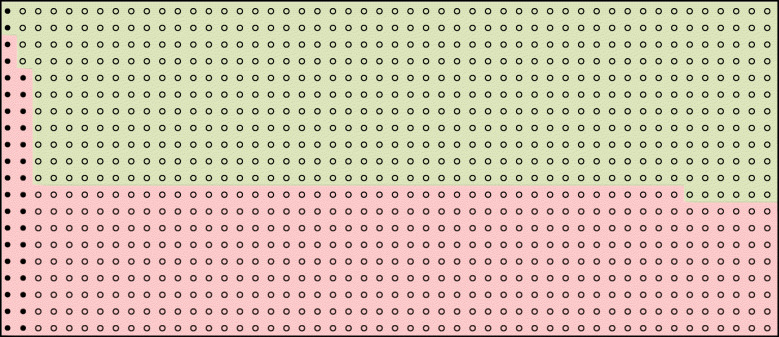
Population diagram to illustrate clinical ramifications of the Canadian Syncope Risk Score for acute management of syncope (cut-point “low risk”). Each circle in the figure represents one person (1000 in total) presenting in the emergency department with syncope, of whom approximately 36 will sustain a serious adverse event (shaded circle) and 964 will not (unshaded circle). Red cells (460) indicate people deemed “at risk” using the risk score with a cut-point of “low risk”. Green cells are people deemed not “at risk”. These natural frequencies are derived from the reported sensitivity of 93%; specificity 56% (for the low-risk cut-point); and prevalence (0.036) in the internally validated model [30]. The internally validated C-statistic for the developed model was 0.88 (95%CI 0.85, 0.90).

The Canadian Syncope Risk Score (CSRS) was developed to help identify patients presenting to the emergency department with syncope who are at risk of developing a serious adverse event, which typically occurs with a prevalence of about 4% [30]. The model was proposed as a risk stratification tool with cut-points signalling very low, low, high and very high risk. The reported internally validated C-statistic for the developed model was 0.88 (95%CI 0.85, 0.90), with sensitivity of 93% and specificity of 53% for the cut-point “low risk”. The rule was summarised by the statement “the tool will be able to accurately *stratify* the risk of serious adverse events among patients presenting with syncope, including those at low risk who can be discharged home quickly”. From the reported sensitivity (93%), specificity (53%) and prevalence (0.036) in the development dataset [30] we estimate the natural frequencies at several cut-points and present a population diagram at one of several reported cut-points for illustration. Figure 1 illustrates how the use of population diagrams can help quantify the implications of using this model at the cut-point “low risk”. This figure illustrates that for this cut-point, whilst of the 540 patients identified as “low risk” by the model only two have a serious adverse event, for every 1000 patients assessed by the model, 460 will be classified as “at risk” of whom only 36 will have a serious adverse event. Therefore, the model used at this cut-point is reasonably able to rule out a serious adverse event, but at the cost of a large proportion of patients undergoing monitoring (i.e. not good at ruling in). Possible consequences of this misclassification are longer stays in hospital for those classified at risk; and a small proportion of patients classified as “low risk” progressing to have a serious adverse event out of hospital. Whilst these might be appropriate trade-offs, they are not obvious when summarising the performance by a C-statistic and sensitivity alone, but become transparent when showing population diagrams. Table 2 presents these natural frequencies across a range of cut-points. For example, if there was a concern that the rule was misclassifying too many people as “at risk” when they would not have the event, increasing the cut-point to 3 for example, would reduce the number classified as “at risk” from 460 to 119; but would increase the number classified as “not at risk” who would have the event from 2 to 12.

**Table 2:**
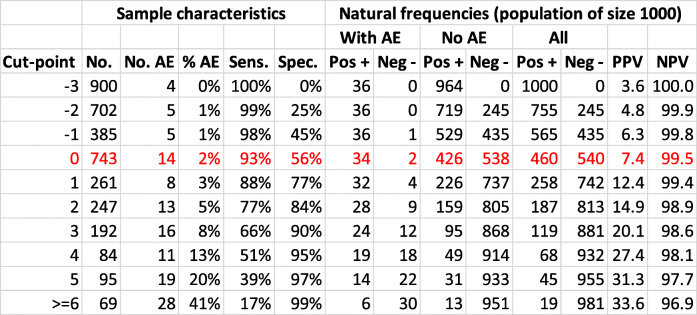
Summary of performance measures across a range of cut-points for the Canadian Syncope Risk Score using natural frequencies

*PPV/NPV* positive (negative) predictive value, *Pos +/Neg −* screened positive or negative for the AE at the given cut-point, *AE* adverse event; assumed prevalence 0.03647; based on a population of 1000; red highlight: cut-point reported in population diagram (Fig. 1). The data under the “sample characteristics” columns are taken from Taljaard [30]

**Table 3:** Case Study 2 the QRISK2 prediction model

**Fig. 2:**
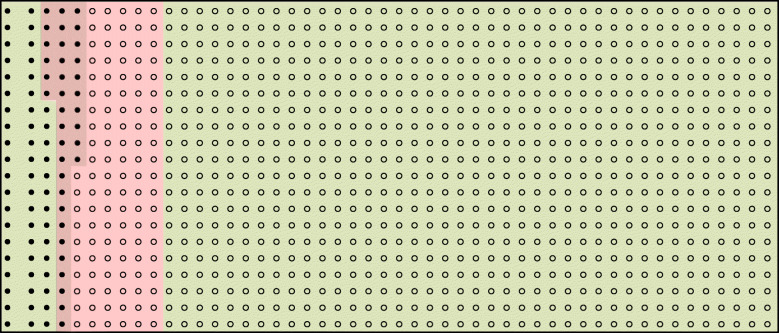
Population diagram to illustrate clinical ramification of the QRISK2 score (cut-point 20%). Each circle (1000 in total) in the figure represents one male between the ages of 35 and 74 years, of whom approximately 90 will have a cardiovascular event over 10 years of follow-up (shaded circles) and 910 will not (unshaded circles). Red shaded cells indicate people deemed “at risk” using the QRISK2 score with a cut-point of ≥20%. Green cells are people deemed “not at risk”. These natural frequencies were derived using the reported natural frequencies in the external validation cohort for males ([4], Table 4). The externally validated C-statistic was 0.77; estimated the sensitivity 40% and specificity 88%; and prevalence of 0.09 over 10 years

The QRISK2 prediction model is a widely endorsed and validated model to assess cardiovascular risk [15]. The QRISK2 model was developed using data from 531 general practices in the UK, with information from 2.3 million patients. The model was developed so as to identify those patients for whom interventions (i.e. statins) or more intensive follow-up may be required. The models are commonly used as part of directive decision-making at a cut-point of 20% predicted risk [15]. The models were reported to perform well, had C-statistics in the region of 0.80, and were subsequently validated in large cohorts [4, 5]. We report natural frequencies for this prognostic rule at the 20% cut-point, derived using information reported in the external validation cohort study for males which had a reported C-statistic of 0.77 [[4], Table 4]. Using data from this validation cohort, it is expected that out of 1000 (male) individuals between the ages of 35 and 74 years, approximately 90 will have a cardiovascular event over a 10-year period and 910 will not (i.e. a prevalence of 0.09) [4]. From data reported in Table 4 of Collins [4], we also estimated the sensitivity of the rule to be 40% and specificity to be 88%. Figure 2 illustrates that, when used at the cut-point of 20%, the prediction rule does not do terribly well in identifying those who will have an event: the rule is correctly able to identify 36 out of the 90 who will have an event, but misclassifies 110 of the 910 individuals who will not have an event as “at-risk”. So, for every person identified as needing treatment, another 3 will be treated unnecessarily and two thirds of those in need of treatment will not be treated. Thus, despite having a C-statistic of close to 0.8, the model does not do terribly well at either ruling-in future events or ruling-out future events [32]. If the extra treatment poses no harm, which might arguably be the case for statin use, then over-treating the low risk patients might not be of concern. Nonetheless, presenting the results using the population diagram allows full implications of potential under and overtreatment to be made transparent. Table 4 presents these natural frequencies across two different assumed prevalence. For example, if the actual prevalence in the population was lower than the assumed 9%, the model would identify slightly fewer at risk; but proportionately more of those at higher risk would be identified as “at risk”

**Table 4:**
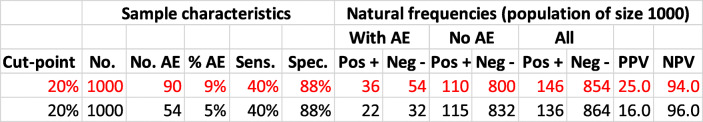
Summary of performance measures across a range of prevalence values for the QRISK2 score (cut-point 20%) using natural frequencies

Values derived from natural frequencies reported in Collins [4] (Table 4) for males, using two different estimates of the underlying 10-year risk

## The importance of considering implications of mis-classification of patients at risk

When a prediction model is intended to be used as a prediction rule, it is important that the implications of imperfect performance of the prediction rule are clear. These implications include two types of misclassification: classifying patients to be at high risk when they will not go on to have the event and classifying patients to be at low risk when they will go on to have the event. The consequences of misclassification are highly contextual, depending on the implications in that particular clinical setting. For example, as illustrated in Case Study 1 (Table 1), the ensuing decision can have serious consequences when misclassifying a patient who is truly at high risk as “not at risk”. On the other hand, the ensuing decision can also have consequences when patients who are not at risk are misclassified as “at risk” (see Case Study 2, Table 3).

The extent of these potential misclassifications should be transparent at all stages of reporting– whether that be when the model is at the development stage, or when the model is at the impact assessment stage. This is because transparent reporting of the extent of the potential misclassification, along with contextual knowledge of the consequences of these misclassifications, can allow the users of these rules to determine how much confidence they place in them. Transparency is important at the development stage because it can help inform the potential impact [26]: for example, in Case Study 1, a prediction rule that clearly misclassifies too many people as low risk might not have been deemed a suitable rule to take forward to an impact study. This transparency is also important at the impact assessment stage—for example, in Case Study 2, if the rule under assessment was known to misclassifying too many people as high risk, then health care providers might re-consider the extent to which they follow the prediction rule. Clear and complete reporting of the performance of prediction rules is thus important at all stages of model development. Reporting results in a transparent way is a form of knowledge translation—where the information on model performance has to be translated by the researchers so it is understood by the intended users, the health professionals. We underscore that our concern is about the communication of the trade-offs or accuracy of the prediction rule at hand, and this is different to communicating the estimated risk from the model [1].

## Common ways of reporting model performance

The Transparent Reporting of a multivariable prediction model for Individual Prognosis Or Diagnosis (TRIPOD) Initiative is a checklist of 22 minimum items for reporting of conduct and results from prognostic model studies ([3] a, [23]). The TRIPOD guidelines recommend that model performance metrics be reported (item 10d) and—whilst not directive in its recommendations—it includes measures of calibration, discrimination, C-statistics, sensitivity and specificity and decision curve analysis [23].

The most commonly reported measure of performance of a prediction model is the C-statistic [22, 23]. Indeed, in a recent review of prognostic models for COVID-19, this was the only measure of predictive performance reported in a set of externally validated models [37]. The C-statistic is a summary measure of performance across all possible cut-points, but it does not quantify the performance at a specific cut-point that may be used to guide management decisions. Because it is a summary performance measure, the C-statistic thus does not convey the performance of the model when used as a prediction rule. Other performance measures which describe the model’s ability to predict risk values which are close to actual levels of risk, called calibration, also summarise overall model performance [26].

To determine how a model performs when used as a prediction rule to risk stratify patients or guide decision-making at recommended cut-points, the performance must be summarised at the given cut-points. Two useful metrics are sensitivity and specificity which describe the rule’s ability to discriminate between those who will and will not have the event at those cut-points [26]. Sensitivity is the ability of the model (at a specified cut-point) to correctly identify those with the event, and specificity is the ability of the model (at a specified cut-point) to correctly identify those without the event. Whilst a chosen cut-point may maximise sensitivity, the trade-off may be poor specificity. Measures such as sensitivity and specificity make the trade-offs at different cut-points transparent. For example, in scenarios such as Case Study 1 (Table 1) where it is important not to miss an event, preference would be given to a cut-point that maximises sensitivity (i.e. a model that is good at “ruling in”). In other scenarios, such as Case Study 2 (Table 3), where there may be potential for over-treatment, it might be important not to falsely diagnose an event and preference would then be given to a cut-point that maximises specificity (i.e. a model that is good at “ruling out”). However, whilst sensitivity and specificity in theory allow the consequences of misclassification to be apparent, there is evidence that these concepts may be misunderstood by health professionals, for example, by confusing sensitivity with the probability of a patient having the event (when in fact it represents the probability of testing positive if the patient has the event) [12, 13].

Alternative ways of summarising a prediction rule’s ability to discriminate (again at specified cut-points) are the positive and negative predictive values (i.e. probability a patient does (or does not) have the event when classified as “at risk” (or “not at risk”) [12]. Positive and negative predictive values also allow the consequence of trade-offs to be transparent at different cut-points. However, whilst positive and negative predictive values prevent the type of misinterpretation commonly observed when interpreting sensitivity and specificity, they are also a conditional probability which can be difficult to interpret [18]. Conditional probabilities are hard to understand because people need to know information on both the probability the person does (or does not) have the event of interest when classified as “at risk” (or “not at risk”)) and contextual information on the likelihood of the event. Negative and positive predictive values thus only communicate one part of this information, but do not convey information on the underlying risk.

## Comparing performance across several prediction rules

Sometimes the performance of several prediction rules are compared. For example, at the derivation stage, the performance of a prediction model might be reported across multiple cut-points, one or two of which are then recommended as the cut-point for implementation in practice (as in Case Study 1, Table 1). Or, sometimes this comparison might be to an existing treatment strategy (such as treat or monitor everyone). Reporting sensitivity and specificity (or negative and positive prediction values) across a range of cut-points allows readers to infer whether a model would work well according to preferences in the particular setting, but is again limited because of the potential for these metrics to be mis-understood.

Decision curves have been proposed as an alternative. Decision curves allow inferences about whether the prediction rule under consideration has a superior net-benefit over alternative strategies (such as treat everyone) [33]. It is recommended that decision curves are presented over a range of cut-points that represent plausible regions of acceptable trade-offs. At any given cut-point, readers can then compare the net-benefit across a set of different strategies (e.g. the prediction rule under consideration and a strategy of treat everyone). The strategy or rule that at any given cut-point maximises the net-benefit is the optimal strategy/rule under the assumption that the trade-offs at that cut-point are acceptable. Yet, decision curves are often viewed as difficult to understand and thus are unlikely to be best suited when conveying information to the health professionals who might use the rule in practice [34]. Furthermore, whilst decision curves are sometimes wrongly assumed to convey information about cut-points that optimise trade-offs, they actually offer a means of comparing net-benefit across different strategies (which might include a prediction rule), but do so under the assumption that the trade-offs are acceptable [17, 34].

## What can be learnt from other areas of communication

There is a large body of research that tells us that when trying to determine if a trade-off is acceptable, people need information about negative and positive predictive values and contextual information on the likelihood of an event [12, 18, 27]. The combination of these two sources of information is known as natural frequencies [13]. For example, when deciding whether to participate in a screening programme for Down’s syndrome, people need to know information on the probability that the baby does (or does not) have Down’s syndrome when classified as “at risk” (or “not at risk”) and the likelihood the baby has Down’s syndrome. This body of work underscores the fact that, to increase understanding amongst patients and members of the public, and consequently facilitate more informed decisions, presenting numerical information using natural frequencies is optimal [27]. Presenting natural frequencies in a visual form has also been shown to increase understanding [24]. Population diagrams (see Case Studies) are one way of visually presenting natural frequencies [18]. Visual presentations have been successfully used in the area of communicating the trade-offs of deciding to participate in screening programmes [7, 18, 27].

Whilst health care professionals tend to have a better ability to interpret statistical information than patients and the public [11], they tend to have some difficulty in interpreting statistical concepts [9, 12, 38]. Furthermore, there is evidence from a systematic review of randomised trials that presenting information using natural frequencies and visual aids increases the understanding of health professionals [13, 35].

## Conveying performance of prognostic rules using natural frequencies and population diagrams

We use two case studies to illustrate how natural frequencies and population diagrams can be useful in helping health professionals decide if a prediction rule has potential to improve treatment or management strategies (Tables 1 and 3). For each case study, we present a population diagram for the prognostic rule at either a recommended cut-point or a cut-point in common use Figs. 1 and 2. Alongside this, for Case Study 1, we illustrate the trade-offs behind the choice of the cut-point using natural frequencies. Cut-points considered to have acceptable trade-offs might then be considered as candidate prediction rules for an impact study. In Case Study 2 (Table 3) we present the natural frequencies at one cut-point only (simply because this is the accepted cut-point used in practice). Presenting the associated population diagram in Case Study 2 allows intended users of the tool (for example health professionals in an impact study) to understand the scope for mis-classification in a rule they have been asked to implement.

Population diagrams are invariably referred to by different names, such as pictograms and decision aids; and can be presented in a variety of ways. Following others, we base the diagrams on a population size of 1000 [10]. Furthermore, we note that the representation of each member of the population might take any of a number of forms, for example a pictorial representation of a person, or as in our population by circles [18, 20]. The diagrams need a coding system that allows two lots of two-way classifications and we follow the format used by Loong [20], whilst noting that alternative ways of presenting, such as scaled rectangular diagrams, might be equally, if not more appealing [21]. Others have suggested that the natural frequency information should be communicated alongside consequences of misclassification [36]. We reiterate that even when presentation of these natural frequencies suggests an apparent well-performing rule, this is not sufficient to indicate if the model should be used in clinical practice, and that all prediction rules should undergo an impact analysis [26]. Indeed, presentation of prediction rules in this way might moreover suggest that the models should be used as an aid in the decision process and not as a substitute or decision rule [19].

Both case studies illustrate how there are trade-offs to be made when using prognostic rules. In Case Study 1, natural frequencies help reveal that when the model is used at the suggested cut-point, whilst it is reasonably able to rule out a serious adverse event, there is a cost—a large proportion of patients are flagged as “at risk” and so would undergo monitoring (i.e. the rule is not good at ruling in). Whilst these might be appropriate trade-offs, they are not obvious when summarising the performance by a C-statistic and sensitivity alone, but become transparent when showing population diagrams. In Case Study 2, for every person identified as needing treatment (i.e. identified as “at risk”), another three will be treated unnecessarily and two thirds of those in need of treatment will not be treated. Thus, despite having a C-statistic of close to 0.8 (actual value 0.77), the model does not do terribly well at either ruling-in or ruling-out future events [32].

## Recommendations for reporting prognostic rules to allow trade-offs to be transparent

When prediction models are recommended to be used as prediction rules there will be trade-offs to be made at the chosen cut-point. These trade-offs should be transparent to the proposed end user—the health professional. Whilst we have not carried out a formal evaluation, these case studies illustrate how in any knowledge translation of prediction rules, population diagrams and natural frequencies are good methods to ensure that the performance of prediction rules can be properly understood. Our goal is to prevent poorly performing rules being adopted in clinical practice because of a misconception that they work well. We advocate not for the replacement of current metrics but rather propose an effective communication tool at the point where researchers have to translate their results to guide clinical decision-making. We make a distinction between (1) providing information in such a way that allows the implications to be compared across multiple cut-points (to facilitate the choice of candidate cut-points that represent a range of acceptable trade-offs in an impact assessment study) and (2) providing information in such a way that allows the implications of the trade-offs at one cut-point to be considered (to facilitate the limitations of a rule when used in clinical practice).

## Data Availability

NA
